# Arsenic, asbestos and radon: emerging players in lung tumorigenesis

**DOI:** 10.1186/1476-069X-11-89

**Published:** 2012-11-22

**Authors:** Roland Hubaux, Daiana D Becker-Santos, Katey SS Enfield, Stephen Lam, Wan L Lam, Victor D Martinez

**Affiliations:** 1 British Columbia Cancer Research Centre, Vancouver, BC, V5Z 1L3, Canada

## Abstract

The cause of lung cancer is generally attributed to tobacco smoking. However lung cancer in never smokers accounts for 10 to 25% of all lung cancer cases. Arsenic, asbestos and radon are three prominent non-tobacco carcinogens strongly associated with lung cancer. Exposure to these agents can lead to genetic and epigenetic alterations in tumor genomes, impacting genes and pathways involved in lung cancer development. Moreover, these agents not only exhibit unique mechanisms in causing genomic alterations, but also exert deleterious effects through common mechanisms, such as oxidative stress, commonly associated with carcinogenesis. This article provides a comprehensive review of arsenic, asbestos, and radon induced molecular mechanisms responsible for the generation of genetic and epigenetic alterations in lung cancer. A better understanding of the mode of action of these carcinogens will facilitate the prevention and management of lung cancer related to such environmental hazards.

## Background

Lung cancer is commonly associated with tobacco smoke exposure. However, lung cancer in never smokers accounts for 10 to 25% of all cases, ranking as the 7th most common cause of cancer-related death [1,2]. As lung cancer in never smokers is thought to develop through molecular pathways different from those induced by tobacco, the study of non-tobacco related carcinogens is fundamental to better understand the biology of lung tumors arising in never smokers [1-5].

**Figure 1:**
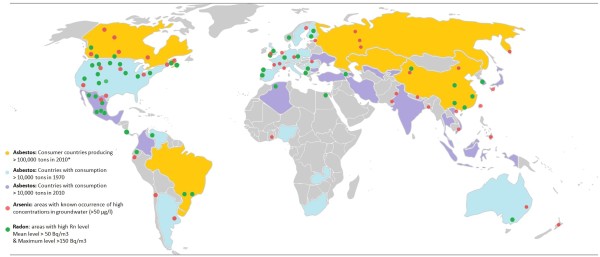
**Worldwide occurrence of asbestos, arsenic and radon.** Regions known to be affected by contamination with asbestos are colored coded in yellow (production >100,000 tons in 2010), blue (consumption >10,000 tons in 1970) and purple (consumption >10,000 tons in 2010). The five largest producers of asbestos (yellow) in 2010 were Russia (1 million tons), China (0.35 million tons), Brazil (0.27 million tons), Kazakhstan (0.23 million tons) and Canada (0.1 million tons). *Zimbabwe produced 0.15 million tons in 2003 but its production was banned in 2004; however, a controversial production revival plan is expected. Countries with current high consumption of asbestos (purple) are distinguished from countries that had previously high consumption prior to the last decades (blue). Grey indicates consumption of less than 10,000 tons per year. Asbestos production and consumption trends (from 1900 through 2003 and for 2010) are provided by the US Geological Survey (USGS) [20,21]. Areas with known occurrence of arsenic in ground water at >50μg/L (red circles) are estimated using information retrieved from the International Groundwater Resources Assessment Centre (USGS) [22]. Areas reported (non-exhaustive) to have high radon levels are depicted by green circles. Radon occurrence was based on ^238^U detected in soil and country radon levels (UNSCEAR, WHO, USGS) [23,24]. Circle placement was determined by approximation; for detailed information, see references as information availability differed from country to country.

Arsenic, asbestos and radon are well known human carcinogens, based on evidence derived from human and animal studies [6,7]. These three agents have been strongly linked to lung cancer development, both in smoker and never smokers [5,8-19]. Due to its wide distribution on a global scale (Figure 1), chronic exposure to these agents poses a significant public health problem. Millions of people, including those who never smoke, are at risk of developing lung cancer induced by arsenic, asbestos and radon.

The carcinogenic effects due to exposure to these elements are well documented [5,8,9]. Table 1 summarizes different sources that provide scientific information linking exposure to these agents with lung cancer and other diseases. These lung carcinogens can induce a wide range of molecular alterations, including genetic (from specific point mutations to genome-wide aberrations) and epigenetic (including alterations in DNA methylation, and microRNA expression) [25]. Considering the relevance of this issue to public health, this article highlights the specific molecular events associated with exposure to arsenic, asbestos and radon as environmental carcinogens driving lung cancer.

**Table 1:** Sources of information on environmental carcinogens associated with lung cancer

**Name**	**Website**	**Description**
The IARC Monographs, International Agency for Research on Cancer (IARC)	http://monographs.iarc.fr/	Compilation of reports about environmental factors that can increase the risk of human cancer: chemicals, complex mixtures, occupational exposures, physical agents, biological agents, and lifestyle factors
Carcinogens, American Cancer Society (ACS)	http://www.cancer.org/Cancer/CancerCauses/OtherCarcinogens/index	Information about environmental carcinogens that can be found at home, work, pollution, medical tests and treatments
Understanding Cancer Series, National Cancer Institute (NCI)	http://www.cancer.gov/cancertopics/understandingcancer/environment	Compilation of slides on environment and its association with cancer
Chemicals of Public Health Concern, World Health Organization (WHO)	http://www.who.int/ipcs/assessment/public_health/chemicals_phc/en/index.html	Information on the 10 chemicals or groups of chemicals of major public health concern
Report on Carcinogens, National Toxicology Program (NTP)	http://ntp.niehs.nih.gov/?objectid=72016262-BDB7-CEBA-FA60E922B18C2540	Congressionally mandated, science-based, public health reports that identify agents, substances, mixtures, or exposures in the environment that may potentially put people in the United States at increased risk for cancer
Science and Technology: Health, Environmental Protection Agency (EPA)	http://www.epa.gov/gateway/science/humanhealth.html	Information on human health impacts associated with environmental exposures
Work-Related Lung Disease (WoRLD) Surveillance System, National Institute for Occupational Safety and Health (NIOSH)	http://www2.cdc.gov/drds/WorldReportData/	Contents on occupationally-related respiratory disease surveillance data.
U.S. Geological Survey (USGS)	http://www.usgs.gov/	Organization that provides impartial information on the health of U.S. environment and the natural hazards
CARcinogen EXposure Canadian Surveillance Project (CAREX)	http://www.carexcanada.ca	Multi-institution research project that combines academic expertise and government resources to generate an evidence- based carcinogen surveillance program for Canada

### Environmental evidence for lung carcinogenesis induced by arsenic, asbestos and radon

#### Arsenic

Arsenic, a naturally occurring metalloid in earth’s crust, is a well-established human carcinogen [7]. Exposure occurs mainly through drinking water, but also via air and food [22,26]. Arsenic contamination has been considered the largest mass poisoning in mankind’s history, since ~160 million people live in regions with naturally elevated levels of arsenic in drinking water [27]. Health effects, including lung cancer, have been documented with chronic exposure at levels below the currently accepted threshold of 10μg/L [3,27-29] – and at such dosage several hundred million individuals would be affected.

Although skin cancer is the most common form of malignancy associated with arsenic exposure, lung, as well as bladder, liver, and kidneys, are other main targets of arsenic carcinogenesis [2,30]. Lung cancer is in fact the main cause of death following chronic arsenic ingestion, and this metalloid is considered as a risk factor for lung cancer in never smokers [2,5,26,31]. Augmented levels of arsenic in drinking water have been associated with an increase in the incidence of lung cancer. In the United States alone, an estimated 5,297 arsenic-related lung cancer cases per year are associated with arsenic exposure [32]. Moreover, arsenic exposure contributes synergistically with other risks factors such as tobacco smoke and history of lung disease [29,33]. The most frequent histological subtypes observed in arsenic-induced lung tumors are squamous cell carcinomas (SqCC) and small cell carcinomas (SCC), which are unusual in tumors arising in never smokers [29,34-37]. Lung SqCC associated with arsenic exposure exhibit unique patterns of genomic alterations, raising the possibility of arsenic-specific oncogenic pathways [37].

#### Asbestos

Asbestos are mineral fibers found naturally in rocks and widely used by industry. Exposure to asbestos fibers, such as chrysotile, amosite, anthophyllite and mixed fibers containing crocidolite, has resulted in a high incidence of lung cancer [6]. Like arsenic, asbestos can act independently or synergistically with tobacco smoke to induce lung cancer [6,38].

Asbestos fibers, with the exception of crocidolite, cause at least twice as many lung cancer deaths than asbestos-related mesothelioma, and these two malignancies combined are responsible for nearly 10,000 deaths per year in the United States [39,40]. The relative risk for developing lung cancer among individuals exposed to asbestos was more than 3 times higher than for non-exposed individuals after controlling for smoking, among other variables [41]. Asbestos-induced effects in the lungs are dose-dependent and are related to the type of fiber inhaled and its composition, such as iron-rich fibers, which are more redox reactive [38,42-45]. Other fibers, such as libby amphibole transition fibers and erionite, although not classified in the asbestos mineral group, have also been implicated in asbestos-associated diseases, suggesting that other thin mineral fibers may have carcinogenic properties similar to those found in asbestos [42]. Even though the current use and management of asbestos is under strict control in most countries, the high latency between exposure and asbestos-related disease development poses a significant public health threat [46]

#### Radon

Radon is a radioactive gas formed naturally by the breakdown of uranium from soils and rocks. Exposure to this gas is estimated to be associated with more than 20,000 lung cancer deaths per year in the United States [47-49]. Radon accounts for more than 50% of the annual effective dose of natural radioactivity exposure [50], affecting not only miners but also the general population as a ubiquitous contaminant of water and indoor environments [50,51].

The relationship between radon and lung cancer has mainly been established from epidemiologic studies of underground miners [52]. Specifically, non-smoking uranium miners in the southwestern United States experienced an increased incidence of lung cancer [53,54]. Further analysis established that up to 70% of lung cancer deaths among uranium miners can be attributed to radon exposure, and the risk of lung cancer among non-smoking miners was up to 3 times higher than in other occupations [48,50,55]. It has been estimated that up to 30% of lung cancer deaths among non-occupationally exposed never-smokers might be linked to indoor radon [48]. The maximum accepted level in most countries is currently 200 Bq/m^3^; however, studies have established an elevated lung cancer risk at radon levels as low as 100 Bq/m^3^[56,57].

### Carcinogenic mechanisms induced by exposure to arsenic, asbestos, and radon

#### Arsenic

**Figure 2:**
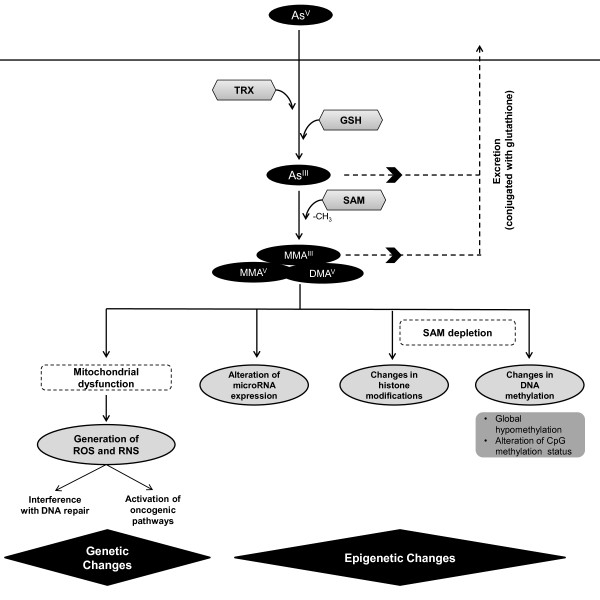
**Arsenic biotransformation drives carcinogenesis.** Arsenic biotransformation occurs through a series of reduction, oxidation, and methylation reactions. Pentavalent arsenic (As^V^) is reduced to arsenite (As^III^), using glutathione (GSH) and thioredoxin (TRX) as electron donors. In the excretion process of this compound, As^III^ is methylated using S-Adenosyl methionine (SAM) as a source of methyl groups; however, this result in generation of arsenic species with higher carcinogenic potential [58-62]. Carcinogenic effects are mostly generated due to this biotransformation process, having effects at genetic and epigenetic levels. Genetic alterations are largely due to generation of reactive oxygen species (ROS) and reactive nitrogen species (RNS), partially derived from arsenic-induced mitochondrial dysfunction. Epigenetic effects, such as changes in DNA methylation patterns have been linked to deprivation of SAM. Changes in miRNA expression and histone modifications have also been reported.

Carcinogenesis of arsenic is related to its biotransformation process. When arsenic enters the body, it induces a series of reduction, oxidation, and methylation reactions (Figure 2) [63]. Pentavalent arsenical species (As^V^ or arsenate) are reduced to trivalent species (As^III^ or arsenite) in a glutathione (GSH)-dependent reaction [64], followed by oxidative methylation resulting in monomethylarsonous acid (MMA^III^), methylarsonate (MMA^V^) or dimethylarsenate (DMA^V^) [65-67]. Some of the methylated metabolites generated in the detoxification process may in fact be more potent carcinogens than inorganic non-methylated species [58-60].

Arsenate interferes with phosphorylation reactions and competes with phosphate transport, while arsenite can react with the sulfhydryl groups of proteins, resulting in inhibition of many biochemical pathways [68]. It is also well established that free radicals are generated during the process of arsenic metabolism [69-73]. By interfering with enzymes that control redox status and glutathione production, arsenic compounds, especially trivalent species, inhibit the protection of cells against oxidative damage [74]. Moreover, arsenic induces a rapid depolarization of the mitochondrial membrane, together with mtDNA deletions and depletions which contribute to carcinogenicity in humans [75,76]. Under these conditions, mitochondria is considered to be the primary site of superoxide anion (·O_2_-) formation [69]. After formation of ·O2- in arsenic-induced oxidative stress, a cascade of secondary reactive oxygen species (ROS), such as hydrogen peroxide (H_2_O_2_) and hydroxyl radical (·OH) is generated [70]. The hydroxyl radical is one of the most impactful ROS and reacts with DNA to produce 8-Hydroxy-2’-deoxyguanosine, a major ROS-induced DNA base-modified product [71,77]. Furthermore, glutathione depletion induced by arsenic may increase its toxicity via ROS-related damage [71,78].

#### Asbestos

**Figure 3:**
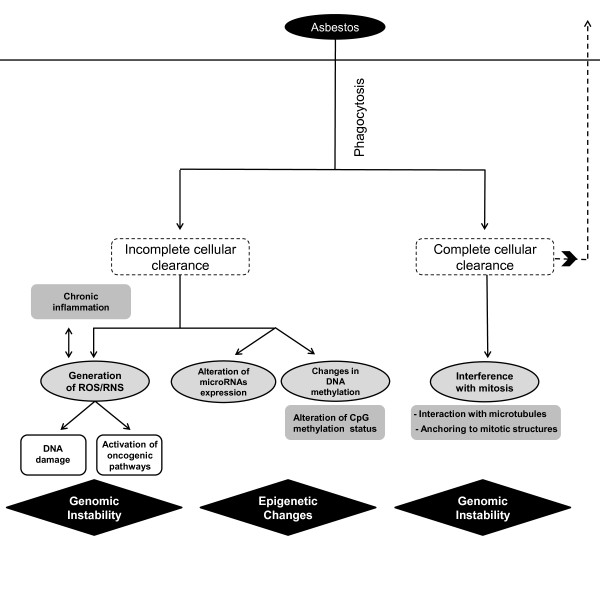
**Mechanisms of asbestos-induced carcinogenesis.** Inhaled asbestos fibers can either be cleared by mucociliary movements and translocations, or undergo phagocytosis [44,82,83]. Fibers not efficiently eliminated by phagocytosis can generate reactive oxygen and nitrogen species (ROS and RNS, respectively) which can lead to generation of DNA single-strand breaks (SSBs) and cell signalling alterations, among other effects. Epigenetic changes, such as alterations in miRNA expression and DNA methylation are also a consequence of incomplete clearance of asbestos fibers. Alternately, fully phagocytized fibers can physically interfere with the mitotic process by interacting directly with microtubules or anchoring to mitotic structures.

Inhaled asbestos fibers longer than 5μm are not efficiently eliminated by phagocytosis. This can induce a cascade of molecular events that lead to fibrosis, inflammation and carcinogenesis [79,80]. On the other hand, fully phagocytized fibers can interfere with mitosis, leading to chromosomal missegregation [81] (Figure 3). The induction of reactive oxygen and nitrogen species upon incomplete phagocytosis of fibers plays an important role in DNA damage [84]. Asbestos induces the release of ROS, including ·O_2_- and H_2_O_2_[79]. Such reactions can be catalyzed on the asbestos fiber surface, and asbestos fibers with high iron content, such as crocidolite and amosite, are capable of generating higher levels of ROS [85]. Similar to arsenic, asbestos also affects mitochondrial DNA and functional electron transport resulting in mitochondrial-derived ROS, which has been shown to induce base oxidation, single-strand breaks, micronuclei, and apoptosis in lung alveolar epithelial cells. [86,87]. Therefore, asbestos carcinogenesis is suspected to occur through creation of an environment of chronic inflammation, and especially through the induction of oxidative stress, a well-known inducer of DNA damage [88]. Lesions at sites of fiber deposition and alterations in gene expression are other relevant mechanisms in asbestos-induced neoplasia in lungs and other target organs [89]. Direct asbestos mutagenicity has also been proposed as a mode of action for asbestos, although this theory requires further study [89,90].

#### Radon

**Figure 4:**
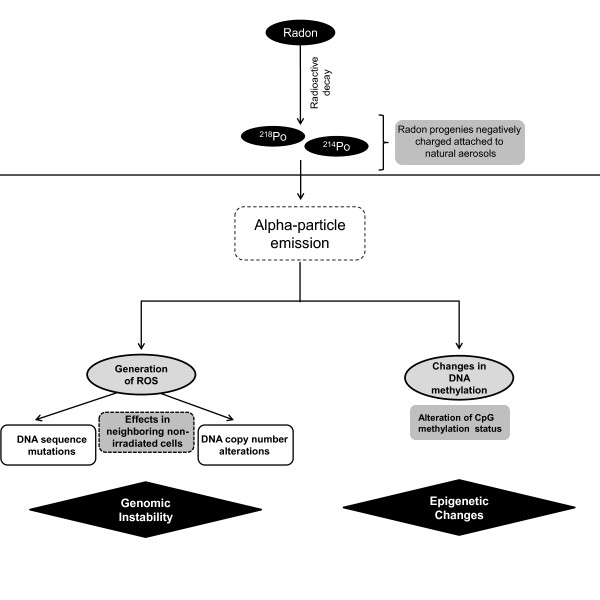
**Radon induces reactive oxygen species through emission of alpha-particles.** Radon decays to radioactive progenies (^218^Po and ^214^Po) that can be inhaled when attached to natural particles in aerosol [50,91]. Once inside the lungs, radon progenies emit alpha-particles that can lead to generation of reactive oxygen species (ROS), eventually resulting in DNA damage not only in the irradiated cell itself, but also affecting neighboring non-irradiated cells [51]. Additionally, changes in DNA methylation are also observed in radon-induced lung tumors.

Although chemically inert, radon decays into active progenies that are electrically charged and can be inhaled when attached to natural aerosols, eventually reaching lung epithelial cells. Once in the lung tissue, deposited radon progeny decays to generate alpha-particles, which damage DNA both directly and through generation of free radicals (Figure 4) [51]. Decay of alpha-particles results in the ejection of electrons from water, generating several reactive species leading to cellular damage by hydroxyl radical attack [92,93]. Cellular damage can also occur in nearby, non-irradiated bystander cells through the release of chemical byproducts by irradiated cells [94]. This ‘bystander’ effect can result in non-linear dose–response damage and underestimation of radon exposure risks [95]. In fact, it has been proposed that lung tissue cellular injury from alpha particles is predominantly due to chromosomal damage in neighboring non-irradiated cells [96]. Moreover, after exposure to alpha-particle radiation, observable levels of cytokines are detected in the supernatants of exposed cells, implying a possible effect of cytokines in radon-induced carcinogenesis [97].

### Genetic and epigenetic consequences in lung tumor genomes due to arsenic, asbestos and radon exposure

The mechanisms discussed above lead to specific genetic alterations including mutations and chromosomal rearrangements, as well as epigenetic changes, meaning mechanisms of gene expression disruption that do not modify the DNA sequence itself, such as DNA methylation, histone modifications and microRNA (miRNA) regulation [98]. Although there have been several reports of specific genetic alterations in lung cancer in never smokers, only a few studies have directly linked such alterations with specific environmental carcinogens.

### Molecular and genetic alterations

#### Arsenic

Moderate levels of arsenic activate the EGFR pathway in human lungs and other target organs of arsenic-carcinogenesis, such as the liver [68,99]. Moreover, arsenic-induced ROS activate the Wnt/β-catenin signaling pathway (which has been shown to promote lung cancer), and stimulate angiogenesis through AKT and ERK1/2 [100-102]. In murine lung tissue, arsenic interferes with expression and protein levels of components of DNA repair machinery, such as APE1, LIG1, OGG1, PARP1 [103].

DMA^V^ has been able to induce lung-specific DNA strand breaks by arsenic-mediated production of ROS in mice [104]. Arsenic has been shown to increase the frequency of micronuclei in cultivated cells from human small airways [105]. Compared to tumors of non-exposed individuals, DNA losses at chromosomal locus 1q21.1 and gains at 19q13.31 and 19q13.33 have been observed lung squamous cell carcinoma (SqCC) in arsenic exposed never smokers [37]. In human small airway epithelial cells arsenic increases expression of cancer related genes and protein levels, such as C-MYC, C-HA-RAS, and C-FOS and decreases β4 integrin protein expression compared with non-exposed cells [105].

#### Asbestos

In lung epithelial cells, asbestos-induced glutathione depletion results in phosphorylation and activation of EGFR, overexpression of different AP-1 proto-oncogenes, and AP-1 transactivation [106]. In human epithelial bronchial cell lines (Beas-2B), asbestos exposure induces a range of gene expression alterations, affecting MAP4K3, CEBPZ, QPCT, FANCG, IGFBPL1, CCL19, MELK, FANCM, and CDKL1 [107]. Asbestos inhalation also causes up-regulation of mRNA levels of matrix metalloproteinase family members in rat lungs, suggesting induction of extracellular matrix remodeling [108]. Mutations in the KRAS and TP53 genes have been detected in animal models human tumors linked to asbestos exposure, although these alterations have not been conclusively associated with asbestos exposure Nelson, 1999 #387, [109,110].

A higher frequency of deletions affecting the P16/CDKN2A locus has been identified in asbestos-exposed non-small cell lung cancer cases compared to unexposed cases, which represent a main gene inactivation mechanism, although no differences were reported linked to smoking status [111]. Changes at chromosomes 5, 8 and 19 have been detected in HBECs transformed by chrysotile asbestos [112]. Additionally, asbestos interferes with chromosomal segregation by interacting directly with microtubules and chromosomes [113-116].

#### Radon

Inhaled particles of radon generate alpha-emissions that cause DNA damage primarily through double-strand breaks and large chromosomal aberrations, mainly deletions and, to a lesser extent, point mutations [117,118]. Specific mutational events have been described in radon-induced lung cancer. Vahakangas *et al*. detected both P53 mutations and deletions in lung tumors from uranium miners. Although the contribution of tobacco smoke cannot be completely ruled out, the most frequent base substitutions associated with tobacco smoking (G:C to T:A transversions), were not identified in that study [119].

Table 2 summarizes the most known genetic alterations observed in lung cancer associated with exposure to these three carcinogens. Specific genetic alterations appear to be prevalent to the exposure to a given carcinogen, for example, DNA losses at chromosomal locus 1q21.1 and gains at 19q13.31 and 19q13.33 are associated with arsenic exposure in never smokers [37].

**Table 2:** Genetic alterations occurring in environmentally induced lung cancer

CNA* **at Locus**	**Carcinogen**	**References**
1q21.1	Arsenic	[37]
2p21-p16	Asbestos	[120] S, [121,122]
Ch.5	Asbestos	[112] CL
5q35	Asbestos	[120] S, [122]
Ch.8	Asbestos	[112] CL
9p21.3 (CDKN2A)	Radon, Asbestos	[123] R, [111]
12p12.1 (KRAS**)	Asbestos	[124]
16p13.3	Asbestos	[122]
17p13.1 (TP53**)	Asbestos	[110] S
Ch.19	Asbestos	[112] CL
19p13.3-13.1	Asbestos	[120] S, [122]
19q13.31	Arsenic	[37]
19q13.33 (SPIB, NR1H2, POLD1)	Arsenic	[37]
22q12.3-q13.1	Asbestos	[122]
Xq28	Asbestos	[120]

* CNA = copy number alteration.

** Sequence mutation.

References include both smokers and non smokers except if indicated (S: smokers only, CL: Cell Lines, R: rat).

### Epigenetic alterations

Specific alterations at the epigenetic level, such as modifications in DNA methylation and microRNA (miRNA) expression patterns, have been associated with arsenic, asbestos and radon exposure. Aberrant methylation of CpG islands in the promoter region of tumor suppressor genes (TSGs) are linked to gene silencing, while deregulation of miRNAs − small, noncoding RNAs species that regulate gene expression − is implicated in diverse human pathologies, including lung cancer (reviewed in [125-128]).

#### Arsenic

Arsenic induces promoter hypermethylation and subsequent transcriptional silencing of tumor suppressors genes, such as P53, CDKN2A and RASSF1A in animal models [129,130]. Chronic arsenic exposure depleted miR-200 levels in human bronchial epithelial cells (HBECs) through increased promoter methylation, and interestingly, re-established expression of miR-200b alone was capable of entirely reversing and preventing arsenic-induced EMT and malignant transformation [131].

#### Asbestos

Epigenetic inactivation of tumor-suppressor genes, such as RASSF1A and CDKN2A (p16) has been observed in lung cancer patients exposed to asbestos [132]. Interestingly, p16 has been found to be inactivated in NSCLC tumors from nonsmokers only through promoter hypermethylation [133]. A recent study has identified an asbestos-associated miRNA signature in lung cancer, where miR-148b, miR-374a, miR-24-1*, let-7d, Let-7e, miR-199b-5p, miR-331-3p, and miR-96, were found to be over-expressed, while miR-939, miR-671-5p, miR-605, miR-1224-5p and miR-202 were under-expressed [134].

#### Radon

Long-term radon exposure has been associated with increased CDKN2A and MGMT promoter methylation among Chinese miners [135]. The locus containing the CDKN2A gene is in fact frequently affected by DNA losses in radon-induced lung tumors in rats [123]. Interestingly, exposure to plutonium, which similar to radon exerts its effects through alpha particles, can induce CDKN2A gene inactivation by promoter methylation [136].

Table 3 summarizes epigenetic changes observed in lung tumors associated with exposure to these three agents.

**Table 3:** Epigenetic alterations occurring in environmentally induced lung cancer

**Type of alteration**	**Carcinogen**	**Gene**	**References**
Hypermethylation	Radon, Asbestos	CDKN2A	[132,135,136]
Hypermethylation	Arsenic	TP53	[129,137]
Hypermethylation	Arsenic, Asbestos	RASSF1A	[130]
Histone Methylation	Arsenic	H3K4, H3K9, H3K27	[138-140]
Histone Hypoacetylation	Arsenic	H4K16	[138,140,141]
Global DNA Hypomethylation	Arsenic	N.A.	[137,142]
miR Downregulation	Arsenic	miR-200	[131] CL
miR Overexpression	Asbestos	miR-148b, miR-374a, miR-24-1*, Let-7d, Let- 7e, miR-199b-5p, miR- 331-3p, miR-96, miR- 17-92	[134] S
miR Downregulation	Asbestos	miR-939, miR-671-5p, miR-605, miR-1224-5p, miR-202	[134] S

Studies concern both smokers and non smokers except if indicated (S: smokers only, CL: Cell Lines).

### Management strategies for radon, arsenic and asbestos exposure

Geological carcinogen mapping plays an essential role in risk management. Examples of geological maps for radon and arsenic can be found on websites from U.S. Environmental Protection Agency as well as from the U.S. Geological Survey (Table 1). Similar maps for Canada are available from the CAREX (CARcinogen EXposure) Canadian surveillance project website ( http://www.carexcanada.ca). Mapping these carcinogens will help to determine occurrence of co-exposure its health consequences, and the urgency for specific management strategies.

Methods for arsenic removal from water include oxidation, precipitation, coagulation, adsorption, nanofiltration, reverse osmosis, and even bioremediation [143]. A cost-effective technique is based on Arsenic Removal Using Bottom Ash (ARUBA) whereby particles of coal bottom ash (a waste material from coal fired power plants), coated with iron hydroxide react with and immobilize arsenic by adsorption and/or co-precipitation. In Bangladesh, ARUBA has been shown to reduce arsenic concentrations in contaminated groundwater to below the Bangladesh safety threshold [144]. Non-viable fungal biomasses of Aspergillus niger coated with iron oxide have also been shown to remove approximately 95% of As(V) and 75% of As(III) from aqueous solutions [145].

The issue of asbestos is more amendable to control since the release of asbestos into the environment originates from human activity. Most developed countries have well documented and regulated management strategies, such as the Italian directives for the remediation of asbestos-cement roofs to be treated prior to disposal on landfill [146]. Some processes are able to eliminate the hazard of these wastes in order to recycle mineral components in new building materials [147,148]. Asbestos-cement wastes are milled in a cyclic process leading to mineralogical and morphological transformations of asbestos while keeping interesting physical properties for building use. On the other hand, two million metric tons of asbestos were consumed in developing countries in 2007, illustrating the need for regulating the use of this carcinogen in developing nations [149].

Strategies against radon rely on radiation detectors and implementation of radon-resistant features; for example, houses in potentially high exposure zones should be equipped with pipes to vent radon gas generated in the ground, and sealed with plastic sheeting and caulking. Ideally, active mitigation techniques involving physical alterations such as sub-slab depressurization should be instigated, as these methods are more effective [150].

## Conclusions

In the next decades, an increasing proportion of lung cancer cases will arise in former or never smokers. While the reduction of environmental carcinogen exposure is certainly a very important cancer prevention issue, understanding the mechanisms of carcinogenesis will facilitate targeted treatment design.

Although tobacco smoke is the major cause of lung cancer, environmental carcinogens, such as arsenic, asbestos and radon play an increasingly important role in this disease, either independently or through additive or multiplicative effects [151,152]. While the number of individuals exposed to these carcinogens is significant, the difficulty to associate tumor cases directly with exposure to these agents (mainly due to the long latency period between exposure and disease onset) may be highly underestimated.

The growing interest in non-tobacco induced causes of lung cancer is reflected in the increasing number of reports describing molecular alterations correlated with exposure to these carcinogens. In this article, we have collected evidence of the involvement of specific molecular mechanisms that can lead to genetic and epigenetic aberrations in lung tumor genomes as a result of exposure to these agents. While sharing a few carcinogenic mechanisms, each agent may induce specific sets of alterations which might affect tumor biology and define tumor behavior, presenting therefore a unique opportunity for developing diagnostic and treatment options. Future research, including the integration of different genetic and epigenetic dimensions, will further the characterization of these etiologically distinct tumors and identify actionable candidates for therapeutic targets.

## Abbreviations

ARUBA: Arsenic removal using bottom ash; Bq: Becquerel; CAREX: CARcinogen exposure canadian surveillance project; DMA^V^: Dimethylarsenate; GSH: Glutathione; H_2_O_2_: Hydrogen peroxide; HBEC: Human bronchial epithelial cell line; MMA^III^: Monomethylarsonous acid; MMA^V^: Methylarsonate; mtDNA: Mitochondrial DNA; RNS: Reactive nitrogen species; ROS: Reactive oxygen species; SAM: s-adenosylmethionine; SCC: Lung small cell carcinoma; SqCC: Lung squamous cell carcinoma.

## Competing interests

The authors declared they have no competing interests.

## Authors’ contributions

RH, DDB, KSSE and VDM designed and collected information for this review. RH and DDB drafted the manuscript. SL and WLL are principal investigators of related projects. All authors have been involved in revision and approved the final manuscript.
